# Comparison of Rebound and Applanation Tonometry in Eyes With Focal Corneal Edema

**DOI:** 10.1111/vop.70075

**Published:** 2025-09-14

**Authors:** Julie A. Kiland, Hannah M. Terhaar, Hannah E. Walleck, Nickolas Chen, Odalys Torné, Kazuya Oikawa, Tina Wahl, Gillian J. McLellan

**Affiliations:** 1 Department of Ophthalmology and Visual Sciences, School of Medicine and Public Health, University of Wisconsin–Madison, Madison, Wisconsin, USA; 2 Department of Surgical Sciences, School of Veterinary Medicine, University of Wisconsin–Madison, Madison, Wisconsin, USA; 3 McPherson Eye Research Institute, University of Wisconsin–Madison, Madison, Wisconsin, USA

**Keywords:** applanation tonometry, corneal edema, feline, glaucoma, intraocular pressure, rebound tonometry

## Abstract

**Objective:**

To determine the effect of corneal edema on intraocular pressure (IOP) readings and their accuracy and precision when obtained with handheld rebound and applanation tonometers.

**Animals and Procedures:**

IOP readings from areas of focal corneal edema were compared to those from clear cornea in 10 eyes of 7 glaucomatous cats in vivo using TONOVET Plus (TVP) and TONOVET (TV01) rebound, and Tono-Pen Vet (TP) applanation tonometers. Four different feline eyes with focal corneal edema were cannulated ex vivo and tonometry readings obtained from clear and edematous cornea at set manometric IOPs from 5 to 70 mmHg.

**Results:**

Rebound tonometry values were 4.0 ± 5.5 (SD) mmHg lower, and TP values were 13.0 ± 5.9 (SD) mmHg higher in regions of edema versus clear cornea in the same eye (*p* = 0.0075). Relative to manometry, TVP and TV01 accuracy and precision were acceptable for clear cornea readings but were negatively impacted by corneal edema. TP-derived readings grossly underestimated high IOPs relative to manometry in both clear and edematous feline cornea and were significantly less accurate than rebound tonometers in clear cornea (*p* = 0.03). All tonometers were less precise in edematous cornea; the TP was significantly less precise than the TV01 (*p* = 0.0419).

**Conclusions:**

Although IOP values obtained with the TVP and TV01 tend to be less accurate and precise in edematous versus clear cornea, rebound tonometry is impacted by corneal edema to a lesser degree than applanation tonometry. In patients with corneal edema, IOP measurements should be obtained from clear corneal regions when possible.

## 1 | Introduction

Ocular hyper- or hypotension is suggestive of ophthalmic diseases, such as glaucoma and uveitis that can lead to partial or complete and irreversible loss of vision [1–5]. Thus, accurate measurement of intraocular pressure (IOP) plays a vital role in the diagnosis and clinical management of ocular diseases in humans and animals. Although the Goldmann Applanation tonometer (GAT) has long been considered the standard for measuring IOP in humans [6], particularly those with ocular hypertension and/or glaucoma, the use of handheld tonometers, such as the Tono-Pen XL applanation and ICare rebound tonometers, has become increasingly popular. These devices are particularly advantageous in veterinary ophthalmology as they are portable, lightweight, and battery operated [6]. Importantly, rebound tonometry requires no topical anesthetic since the tonometer probe contacts the cornea for only microseconds [7].

The Tono-Pen Vet applanation tonometer (TP; Reichert), the TonoVet rebound tonometer (TV01; ICare, Finland), and an updated version of the TV01, the TONOVET Plus (TVP; ICare, Finland), are commonly used in clinical veterinary ophthalmology and in experimental animals to measure and monitor IOP. The TV01 and TVP have the same basic configuration; however, the TVP adds species-specific settings for cats (feline’) and rabbits (lapine’) to the original TV01 settings for dogs and horses. In addition, the TVP provides features notifying the user whether the device is being used at the correct distance and/or orientation in relation to the ocular surface. Numerous studies validating the TV01, TP, and TVP have been reported in a variety of species including dogs [8–16], cats [17–20], horses [15, 21], rabbits [22–24], macaques [25], and others [26–29]. However, previous studies have shown that alterations in corneal thickness and biomechanical properties, including corneal abnormalities such as edema, scarring, and pigmentation, can affect IOP measurements obtained with both rebound and applanation tonometers in humans and in animals [14, 22, 30–34] Determining the effect of corneal disease on the accuracy of IOP measurements taken via rebound and applanation tonometry is clearly of immense importance in the clinical diagnosis and monitoring of patients with ocular diseases that affect IOP, including secondary and syndromic glaucomas and posttraumatic uveitis, in which there may be concurrent focal or diffuse corneal pathology. A study in the veterinary literature that compared IOP measurements acquired from focal areas of diseased versus clear cornea in the same eyes of domestic dogs and cats found variances in IOP values from −6 to 16 mmHg and −7 to 20 mmHg with the TonoVet and Tono-Pen Vet, respectively, that in turn varied substantially with the type of corneal pathology. However, focal edema, in the absence of glaucoma or chronic keratitis, was implicated as the cause of focal corneal disease in only one eye (with corneal dystrophy) in the study. The clinical cases included in that study were individual dogs or cats with chronic keratitis due to a wide range of underlying etiologies that included corneal sequestrum, chronic superficial keratitis, prior ulceration, lipidosis, corneal exposure and/or keratoconjunctivitis sicca [14]. While the study clearly illustrated the profound effect that corneal disease has on tonometry readings, variability in underlying etiopathology between eyes, small number of eyes with each diagnosis, and lack of direct manometric comparisons meant that the effect of disease on tonometer accuracy and precision could not be determined [14].

The current study sought to address gaps in knowledge by determining the accuracy and precision of IOP values obtained with the TonoVet and TONOVET Plus rebound tonometers and the Tono-Pen Vet (TP) applanation tonometer in feline eyes with focal corneal edema, comparing tonometry values obtained in focal areas of edema to those obtained from areas of clear cornea in the same eyes.

## 2 | Materials and Methods

### 2.1 | Animals

**FIGURE 1 |:**
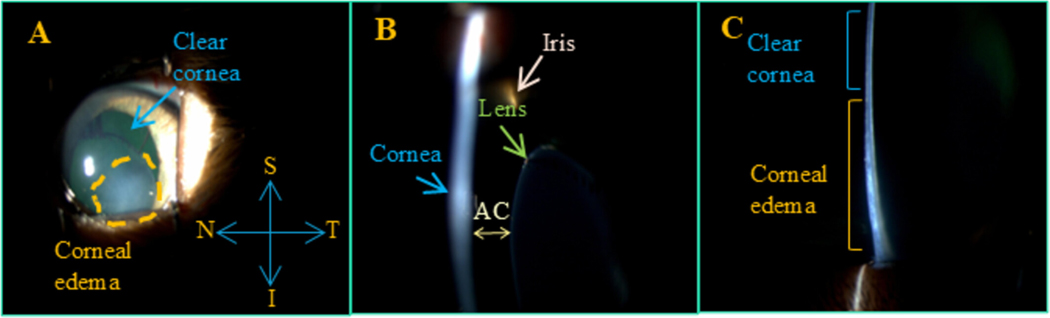
Representative photos obtained with a slit lamp showing area of focal corneal edema on the inferior area of the cornea (A), relationship of lens and iris to cornea (B), and difference in thickenss between clear cornea and edematous cornea in one eye of one cat. S=superior, T=Temporal, N=Nasal, I=Inferior; AC=Anterior Chamber.

A total of seven glaucomatous homozygous *LTBP2*-mutant cats [35] (10 eyes), ranging in age from 6 months to 4 years, were studied in vivo. All were purpose-bred animals within a research colony maintained as a model for the study of glaucoma related to spontaneous *LTBP2* mutation [36, 37]. Animals were maintained under controlled laboratory conditions with a 12 h light: dark cycle, and all cats were acclimated to their surroundings, to frequent handling, and to weekly IOP measurements taken with the TV01 rebound tonometer for a minimum of 4 weeks before beginning the study. Animals were gently restrained in a seated or sternal position for IOP measurements. No attempt was made to randomize the order of eyes, with measurements typically taken in the right eye first followed by the left. Although lens instability and corneal opacity are not consistent features of glaucoma in this pedigree, eyes selected for study had pronounced, focal, paraxial corneal edema related to endothelial damage by previous anterior lens luxation or Descemet’s membrane breaks. (Figure 1A–C) An additional four different glaucomatous *LTBP2*-mutant cats (4 eyes) with focal corneal edema, aged 5.5 months to 2.6 years, were tested opportunistically, ex vivo, within 5 h *postmortem* following humane euthanasia for reasons unrelated to the current study. Eyes were immediately enucleated and kept refrigerated in a sealed container at 4°C between euthanasia and testing. All experiments were performed with the approval of the University of Wisconsin-Madison Institutional Animal Care and Use Committee and in accordance with the ARVO Statement for the Use of Animals in Ophthalmic and Vision Research.

### 2.2 | Clinical Evaluation In Vivo

#### 2.2.1 | TonoVet, TONOVET Plus, and Tono-Pen Vet in Cats With Focal Corneal Edema

Intraocular pressure (IOP) measurements were obtained by a single observer using the TV01, TVP, and TP consecutively. Triplicate IOP readings were taken with each tonometer in each eye on an area of focal corneal edema followed by an area of clear cornea. TV01 readings were obtained first, followed by the TVP and TP. One drop of topical anesthetic (Proparacaine Hydrochloride 0.5%, Bausch & Lomb) was instilled in each eye ~60 s prior to TP readings [38]. The TVP was set to the “feline” mode and the TV01 was set to the “d” (dogs and cats) mode based on previous studies [18, 19]. The TP does not have species-specific settings. Readings were recorded if they were deemed acceptable, as indicated by a green ring on the TVP display; an absent or low bar on the TV01 display, or the appearance of a bar under the < 5% SD or < 10% SD indicator on the TP display. According to the TVP and TV01 instruction manuals, acceptable measurements are defined as those that have a standard deviation ≤ 2.5 of the mean of four probe deceleration values (after the algorithm discards readings acquired with the highest and lowest deceleration values). The area and extent of corneal edema differed among the cats, so we could not readily acquire measurements from a consistent area of clear cornea between the eyes/cats. Corneal edema was most often present in the central and inferior and paracentral cornea, whereas clear areas were typically located in the nasal and/or temporal cornea, which is where IOP measurements were obtained. Although different eyes/cats had different areas of clear cornea, repeated measurements using each tonometer type were acquired from the same area of clear cornea within the same eye/cat. Previous studies have shown either no or minimal differences between readings taken on the central versus the temporal and/or nasal corneal regions with the ICare rebound tonometer in humans and that IOP readings taken from the nasal and temporal cornea can be used as a reliable estimate of IOP when readings cannot be taken from the central cornea [31, 39].

### 2.3 | Manometric Evaluation Ex Vivo

#### 2.3.1 | TonoVet, TONOVET Plus, and Tono-Pen Vet vs. Manometry in Eyes With Focal Corneal Edema

Intraocular pressure readings were obtained in each eye at manometrically determined IOP values using the three tonometers according to previously published methods [29]. Briefly, the anterior chamber of each eye was cannulated with two 25-gauge needles inserted through the corneal limbus: one at the 10 o’clock position and the other at the 2 o’clock position. There was no grossly visible corneal distortion, but the effect of the needle placement on corneal curvature was not directly measured. The needle positioned at the 10 o’clock position was connected to a pressure transducer (DTXPlus, Argon Medical Devices) and to a continuous physiologic recorder (Dash 4000 Pro, GE Healthcare). The transducer line was filled with lactated Ringer’s solution (LRS) and calibrated to zero using a mercury manometer connected via polyethylene tubing. The second needle was connected via polyethylene tubing to a 500 to 1000 mL bag of LRS. The pressure in the anterior chamber was set by raising and lowering the height of the fluid bag. The needles were placed so that corneal curvature was not affected, and the corneal surface was continuously irrigated to prevent desiccation. The pressure was initially set at 5 mmHg and then increased in 5 mmHg increments to 40 mmHg and then in 10 mmHg increments to 70 mmHg. Triplicate IOP readings were obtained in each eye using the TV01, TVP, and TP in an area of focal edema and then in an area of clear cornea at each pressure setting. The same unmasked observer (JK) took all measurements to avoid the potential for measurement discrepancies due to interoperator differences in device handling technique. Readings that were deemed acceptable were recorded according to the parameters defined for clinical evaluation above.

### 2.4 | Statistical Analysis

Microsoft Excel and GraphPad Prism v9 (GraphPad Inc.) were used for statistical analyses. IOP readings obtained from both clear cornea and regions of focal edema were compared by paired *t*-test for each tonometer. Linear regression analyses and ordinary one-way ANOVAs, followed by Tukey–Kramer multiple comparisons test, were performed comparing the slope and *R*^2^ values between devices for ex vivo analyses. Determination of precision was based on the *R*^2^ value obtained from the linear regression equation generated for each device; the higher the *R*^2^ value, the better fit to the regression line, indicating more repeatable/precise measurements. Bland–Altman plots were used to evaluate the agreement between IOP values from areas of corneal edema versus clear cornea for each tonometer. A *p* value < 0.05 was considered statistically significant.

## 3 | Results

### 3.1 | Clinical Evaluation of the TonoVet, TONOVET Plus, and Tono-Pen Vet in Cats With Focal Corneal Edema

**FIGURE 2 |:**
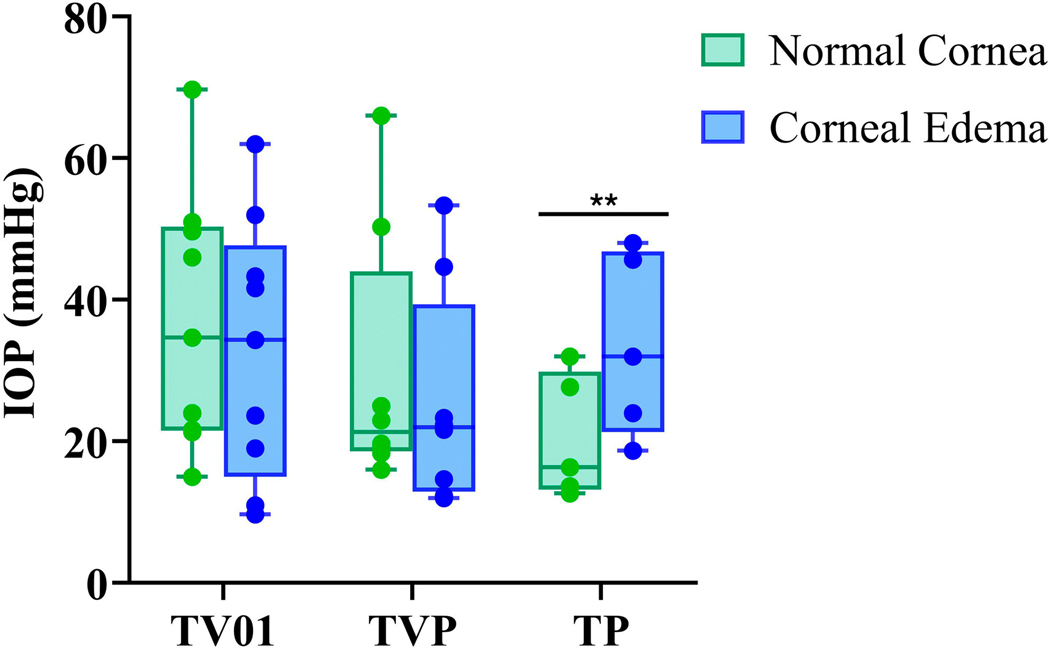
Box and Whiskers plot showing mean IOP values obtained in 7 cats with the TV01, TVP, and TP in regions of corneal edema and normal cornea in the same eye. IOP values obtained by the TP from areas of corneal edema were significantly higher compared to areas of clear cornea. **p=0.0075.

IOP measurements taken with TV01 and TVP were lower by a mean of 4.04 ± 5.45 (SD) mmHg (*p* = 0.057) and by 4.17 ± 5.68 (SD) mmHg (*p* = 0.077), respectively, in regions of focal corneal edema versus clear cornea in the same eye (Figure 2). These differences were not statistically significant. IOP values obtained with the TP were significantly higher, by a mean of 13.0 ± 5.9 (SD) mmHg, in regions of focal corneal edema versus clear cornea (*p* = 0.0075) (Figure 2, Table 1).

### 3.2 | Manometric Evaluation Ex Vivo of the TonoVet, TONOVET Plus, and Tono-Pen Vet in Cats With Focal Corneal Edema

**FIGURE 3 |:**
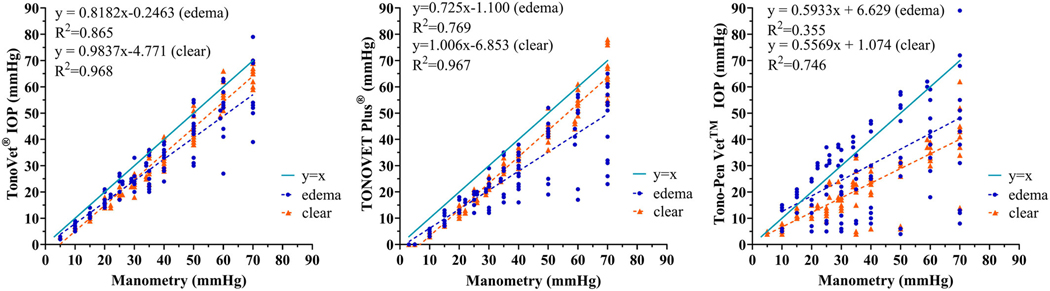
IOP readings obtained with the (A) TV01, (B) TVP, and (C) TP were strongly linearly correlated to manometric IOP. All three tonometers underestimated IOP readings obtained on clear and edematous cornea relative to manometry. Readings obtained on clear cornea were significantly more accurate with the TV01 (p=0.0353) and tended to be more accurate with the TVP compared to the TP. There was no significant difference in accuracy or precision between the TVP and TV01 whether readings were taken on clear or edematous cornea. The TP grossly underestimated IOP on both areas of clear cornea and corneal edema, though IOP values obtained with the TP from areas of corneal edema were higher than those from clear cornea as corneal edema lessened extent of IOP underestimation by the TP. Linear regression lines are shown as dashed and y=x solid line shown for reference. Some data points are super-imposed.

**FIGURE 4 |:**
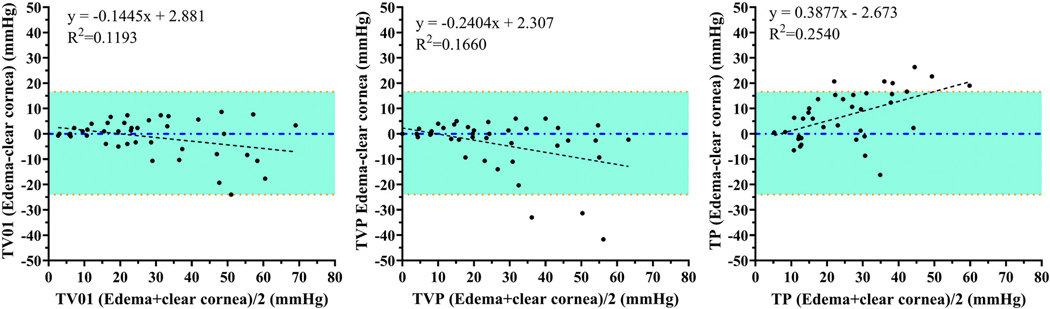
Bland-Altmann plots indicate agreement between readings taken with the TV01 (A), TVP (B), and TP (C) on edematous vs. clear cornea. Agreement between readings taken on edematous vs. clear cornea was closest with the TV01. Linear regression lines are shown as dashed. Upper and lower red dotted lines indicate 95% limits of agreement. Some data points are super-imposed.

All tonometers yielded IOP values that showed relatively strong linear trends on regression analysis, plotted against manometric IOP. All three underestimated IOP for readings obtained from both clear and edematous cornea relative to manometry. Accuracy and precision were acceptable for the TV01 and TVP when readings were obtained from clear cornea. Readings obtained from edematous cornea were less precise with all three tonometers when compared to those obtained from clear cornea, though the difference was not significant. The TV01 was significantly more accurate and the TVP tended to be more accurate compared to the TP when readings were taken on clear cornea. There was no significant difference in accuracy or precision between the TVP and TV01 whether readings were taken from clear or edematous cornea, though corneal edema increased underestimation of IOP for both. The TP grossly underestimated IOP on both clear and edematous cornea. However, IOP values obtained with the TP from areas of corneal edema were higher than those from clear cornea, as corneal edema lessened the extent of IOP underestimation by the TP. (Figure 3A–C, Table 2). Agreement between readings taken on edematous versus clear cornea was closest with the TV01 (Figure 4A–C).

## 4 | Discussion

In the current study, IOP readings obtained in feline eyes using rebound tonometry tended to be more accurate and precise than those obtained via applanation tonometry ex vivo. In addition, rebound tonometry appeared to be impacted by corneal edema to a lesser extent than applanation tonometry. The TP substantially underestimated IOP values taken from both clear and edematous corneas. This is consistent with results of previous studies showing underestimation of IOP by the TonoPen XL versus manometry in normal and glaucomatous cat eyes [18–20]. TP measurements obtained in vivo were significantly higher from areas of corneal edema versus clear cornea, and ex vivo TP values obtained from areas of corneal edema tended to be higher than those from clear cornea as well.

It has been established that increasing corneal thickness is positively correlated with increased IOP readings obtained by applanation tonometry [40, 41]. In humans, Goldmann applanation tonometry has been shown to overestimate IOP in eyes with mild corneal edema likely due to an increase in central corneal thickness (CCT) as well as potential effects on corneal rigidity [42, 43]. A study in human eyes with mild corneal edema following cataract surgery suggested that the extent of corneal edema, in addition to the level of IOP, has an impact on whether applanation tonometry underestimates or overestimates IOP [42], however, an earlier study found no correlation between the degree of corneal edema and resultant IOP values obtained with applanation tonometry [44]. Unlike applanation tonometry, rebound tonometry measures IOP based on rebound velocity of a magnetized spherical probe against the surface of the eye, with higher pressures corresponding to faster rebound and shorter duration of contact [12]. Thus, in eyes with corneal edema, it is speculated that IOP measurements taken by rebound tonometry might be lower due to corneal edema dampening impact and retarding rebound of the probe [42].

A pilot study comparing the accuracy and precision of the ICare rebound tonometer (human equivalent to the TonoVet) and applanation tonometer against a digital manometer in human cadaveric eyes with extreme, diffuse corneal edema reported that both the ICare and applanation tonometers underestimated manometric IOP at pressures > 10 mmHg, and that the applanation tonometer performed better than the ICare, particularly at higher pressures [45]. However, only two human cadaveric eyes were included in that study, and no comparisons were made between readings obtained from areas of corneal edema and readings obtained from clear cornea due to the diffuse nature of the corneal edema in these eyes.

One limitation of our study is that tonometers were only evaluated in eyes with corneal edema and not in eyes with other types of corneal pathology, such as pigmentation, scarring, and vascularization. The previously described study by von Spiessen et al. evaluated IOP measurements taken in 29 eyes of dogs and cats affected by a very diverse range of focal corneal pathologies including pigmentation, scarring, lipidosis, edema, vascularization, sequestra, or ulceration, and found that measurements on diseased versus normal cornea resulted in deviations in IOP from −6 to +16 mmHg (average + 2.1 mmHg) and from −7 to +20 mmHg (average + 3.2 mmHg) for the TonoVet and Tono-Pen, respectively [14]. This study clearly highlighted that readings obtained with both rebound and applanation tonometers vary greatly depending on the type of corneal abnormality. However, there were often only one or a few eyes with a particular corneal pathology, and many eyes with chronic keratitis had multiple contributing corneal pathologies such as fibrosis, vascularization, and edema. Additionally, direct manometry was, understandably, not performed in that clinical study, so conclusions could not be drawn regarding the influence of individual pathologies on the accuracy and precision of TonoVet and Tono-Pen measurements. While our studies definitively established the effect of corneal edema on tonometry values and their accuracy and precision, future studies are needed to evaluate the effects of additional types of corneal pathologies, such as scarring, vascularization, sequestra, and keratoconus on applanation and rebound tonometry.

Another limitation is that it was not possible to consistently measure corneal thickness or perform anterior segment OCT in all the affected eyes, due to the opportunistic nature and timing of the studies, which limited access to clinical equipment. It is acknowledged that more in-depth characterization of the corneal pathology present in our cases could have provided greater in-sight and more comprehensively addressed existing controversy regarding the extent to which corneal edema impacts tonometry values [42, 44]. Thus, future studies investigating the direct quantitative relationship between corneal edema and its effects on corneal thickness, corneal material and biomechanical properties (i.e., hysteresis, stiffness, deformability), and consequent effects on tonometry values are needed.

In summary, despite the limitations of our study design, we were able to demonstrate that rebound tonometry tended to be more accurate and precise in areas of clear cornea versus areas of focal corneal edema in feline eyes in the current study and correlated better with manometric IOP than the applanation tonometer used. This suggests that rebound tonometry is an appropriate and preferred method for measuring IOP in eyes with focal corneal edema. As IOP values obtained with the TV01 and TVP in regions of corneal edema tend to be substantially less accurate and precise compared to those obtained from clear cornea, IOP measurements should be obtained from adjacent clear corneal regions whenever possible.

## Figures and Tables

**TABLE 1 |:** Comparison of IOP values acquired from regions of edematous cornea and adjacent clear cornea, with three veterinary tonometers.

Tonometer	Corneal edema	Clear cornea	Difference
TonoVet	32.96 ± 18.36	37.00 ± 18.18	−4.04 ± 5.45
TONOVET Plus	25.54 ± 15.33	29.71 ± 18.27	−4.17 ± 5.68
Tono-Pen Vet	33.67 ± 12.95	20.47 ± 8.79	13.2 ± 5.91*

*Note:* Values are mean ± SD of triplicate IOP (mmHg) readings.

* *p* = 0.0075.

**TABLE 2 |:** Accuracy and precision of three veterinary tonometers on clear and edematous cornea.

	Edema			Clear		
Tonometer	Slope	Intercept	*R*^2^ (precision)	Slope	Intercept	*R*^2^ (precision)
TonoVet	0.818	−0.246	0.865	0.984^a^	−4.771	0.968
TONVET Plus	0.725	−1.100	0.769	1.006	−6.853	0.967
Tono-Pen Vet	0.593	6.629	0.355	0.557^a^	1.074	0.746

a *p* = 0.0353.

## Data Availability

The data that support the findings of this study are available from the corresponding author upon reasonable request.
